# Sarcopenia and inflammatory markers in early-stage breast cancer: a retrospective case-control study of NLR, MLR, RDW-CV, and HRR in middle-aged and elderly women

**DOI:** 10.3389/fsurg.2026.1884306

**Published:** 2026-08-12

**Authors:** Yu Liu, Xinyu Zhai, Zhongyu Wang, Wei Jia

**Affiliations:** 1 Hebei Medical University, Shijiazhuang, Hebei, China; 2 Department of Breast Surgery, Hengshui People’s Hospital, Hengshui, Hebei, China; 3 Hebei University, Baoding, Hebei, China

**Keywords:** early-stage breast cancer, female, inflammatory markers, middle-aged and elderly, sarcopenia

## Abstract

**Objective:**

To investigate the association between sarcopenia and inflammatory markers in middle-aged and elderly women with early-stage breast cancer.

**Methods:**

Data collection was performed between April 18 and April 30, 2026. We retrospectively collected data from 263 middle-aged and elderly female patients with early-stage breast cancer who were newly diagnosed and treatment-naïve in the Department of Breast Surgery at Hebei Medical University Affiliated Hengshui People's Hospital (Harrison International Peace Hospital) between 01/09/2024 and 31/01/2026. Patients were classified into the sarcopenia and non-sarcopenia groups based on the presence of concurrent sarcopenia. Baseline patient characteristics, clinical indicators, and inflammatory markers were collected. Multivariate logistic regression was used to analyze the relationship between sarcopenia and inflammatory markers. The predictive value of NLR for sarcopenia in early-stage breast cancer was evaluated using ROC curve analysis.

**Results:**

Compared with the non-sarcopenia group, the sarcopenia group were significantly older, neutrophils, RDW-CV, PLR, NLR, MLR, and were more likely to be postmenopausal (*P* < 0.05). Conversely, body weight, BMI, alanine aminotransferase, prealbumin, cholinesterase, lymphocytes, hemoglobin, red blood cell count, and HRR were significantly lower in the sarcopenia group (*P* < 0.05). Multivariate logistic regression revealed that increased age, elevated hemoglobin, and elevated NLR were independent risk factors for sarcopenia (*P* < 0.05), while higher BMI, RDW-CV, MLR, red blood cell count, and HRR were independent protective factors. ROC curve analysis indicated that the area under the curve of NLR for predicting sarcopenia was 0.772.

**Conclusion:**

An elevated NLR was independently associated with sarcopenia in middle-aged and elderly patients with early-stage breast cancer and has reference value for predicting sarcopenia in this population, which may serve as an inexpensive preliminary screening marker.

## 1 Introduction

Breast cancer is one of the most common malignancies among women worldwide, with its incidence and mortality ranking high on a global scale (1). In addition to the disease burden itself, middle-aged and elderly patients also experience significant impairment in their quality of life. With continuous advances in treatment modalities, patient survival has been significantly prolonged; however, this clinical attention has increasingly shifted toward managing treatment-related complications. Complications of breast cancer include upper limb lymphedema, leukopenia, and skeletal and muscular impairments. Among these, musculoskeletal impairment has become an increasingly recognized clinical concern.

Sarcopenia, an age-related syndrome, is characterized by reduced muscle mass, decreased muscle strength, and decline in physical function, and is associated with frailty and increased risk of falls (2). Recent studies indicate that the prevalence of sarcopenia in the general population ranges from 5% to 10%, with a significant increase as age advances (3). In breast cancer patients, the reported prevalence of sarcopenia is notably higher, reaching 12.2%–73.2% (4). Accumulating evidence suggests that sarcopenia may be triggered by inflammatory cytokines released by the tumor, and is linked to tumor-associated inflammation, side effects of chemotherapy, and malnutrition (5). Moreover, sarcopenia is associated with poorer clinical prognosis in breast cancer patients. The coexistence of sarcopenia and breast cancer not only reduces treatment tolerance, impairs performance status and quality of life, but also increases the risk of chemotherapy toxicity, postoperative complications, falls, and fractures (6). If not effectively managed, it may further lead to functional decline and raise the risks of infection, cardiovascular events, and even mortality (7).

Chronic inflammation plays a central role in the development, progression, and recurrence of breast cancer by affecting the tumor microenvironment. A persistent inflammatory state fosters a pro-cancer environment by enhancing communication between cancer cells and surrounding stromal cells (such as fibroblasts and immune cells), thereby promoting tumor progression (8). In chronic conditions like cancer, inflammatory factors released by tumors or inflammatory cells trigger a systemic inflammatory response that directly interferes with skeletal muscle metabolism. This leads to increased muscle protein breakdown, reduced synthesis, and may activate apoptosis and regeneration, ultimately resulting in muscle atrophy and functional loss (9). Therefore, identifying readily available biomarkers in clinical practice that can reflect the body's systemic inflammatory status is of considerable clinical value for the early identification of individuals at high risk for sarcopenia. While traditional inflammatory markers like C-reactive protein and IL-6 are valuable, their detection costs are relatively high and they are susceptible to short-term factors. In recent years, novel inflammatory markers derived from routine peripheral blood tests have been widely studied in oncology due to their advantages of being cost-effective, convenient, and highly reproducible. Among them, the NLR reflects the imbalance between innate immunity and adaptive immunity (10). The MLR reflects the activation level of the monocyte-macrophage system, a major source of pro-inflammatory cytokines (11). Additionally, RDW-CV is not only an indicator of erythrocyte volume heterogeneity but is also increasingly recognized as a sensitive marker reflecting chronic inflammation, oxidative stress, and malnutrition (12). Accordingly, the HRR, calculated by combining hemoglobin and RDW-CV, is a composite indicator that can simultaneously assess anemia and inflammatory status, potentially providing a more comprehensive reflection of the body's pathophysiological disturbances (13).

While these inflammatory markers have shown great promise in predicting the prognosis of various solid tumors, research on their correlation with breast cancer patients, particularly within the specific population of middle-aged and elderly women with early-stage breast cancer, remains relatively scarce. The physical condition of early-stage breast cancer patients before radical treatment can better reflect tumor-related pathological changes and exclude the interference of subsequent therapies. Therefore, this study aims, through a retrospective cross-sectional analysis, to systematically investigate the correlation between the occurrence of sarcopenia and a series of blood inflammatory markers, including NLR, MLR, RDW-CV, and HRR, in middle-aged and elderly women with early-stage breast cancer prior to initial treatment. It also seeks to evaluate the potential clinical value of these readily accessible markers as early screening tools. The findings aim to provide evidence-based medical support for the subsequent identification and management of middle-aged and elderly women with early-stage breast cancer complicated by sarcopenia, and for formulating individualized strategies for nutritional intervention, rehabilitation training, and anti-inflammatory therapy.

## 2 Materials and methods

### 2.1 Study subjects

Using the hospital electronic medical record system, we retrospectively collected 263 cases of early-stage breast cancer in middle-aged and elderly women who were admitted for the first time and had not received prior treatment in the Department of Breast Surgery at Hebei Medical University Affiliated Hengshui Hospital (Harrison International Peace Hospital) between 01/09/2024 and 31/01/2026. Inclusion criteria: (1) Middle-aged and elderly women; (2) Pathologically confirmed primary early-stage breast cancer (TNM stage I-II according to the 8th edition of the American Joint Committee on Cancer staging system); (3) Had not received any form of anti-tumor treatment prior to admission, including neoadjuvant chemotherapy, radiotherapy, endocrine therapy, targeted therapy, or immunotherapy; (4) Completed abdominal computed tomography (CT) scans and all required laboratory tests after admission. Exclusion criteria: (1) Concurrent active malignancies in other sites or a history of malignancy; (2) Presence of definite acute infectious diseases or being in the acute phase of infection; (3) Suffering from active autoimmune diseases or receiving immunosuppressant therapy; (4) Presence of severe heart, liver, or kidney dysfunction; (5) Concurrent hematological diseases affecting peripheral blood cell counts; (6) Severe lack of clinical data, imaging data, or laboratory data, making effective assessment impossible.

The research protocol was approved by the Ethics Committee of Hengshui People's Hospital, affiliated with Hebei Medical University (Ethics Approval Number: 2026036). Given the retrospective design of this study and the anonymization of all data, it poses no additional risk to patients.

### 2.2 Diagnosis of sarcopenia and grouping

In recent years, a consensus has been reached in the medical community that measuring the skeletal muscle cross-sectional area at the third lumbar vertebra (L3-SMA) level on abdominal CT scans is the preferred method for diagnosing sarcopenia (14). Furthermore, research has confirmed that the ratio of muscle area at the third lumbar vertebra level to the square of height (L3-SMI) can not only accurately reflect muscle wasting but also independently predict patient survival time (15). In fields such as oncology, cirrhosis, and gastrointestinal diseases, L3-SMI has been proven to be an important indicator for assessing sarcopenia and its prognosis (16, 17). For example, a 2023 study noted that CT remains the gold standard for measuring muscle mass, as it provides cross-sectional images for accurate assessment and is not affected by tissue edema, ascites, or intra-abdominal tumors. The study also mentioned that a single slice at the L3 level is the most reliable method for assessing sarcopenia (18).

Building on this, and to align with the latest research specific to the Chinese population, we adopted an SMI ≤ 34.90 cm²/m² as the diagnostic threshold for sarcopenia in women (19). According to criterion, all patients were divided into two groups: the Sarcopenia group and the non-sarcopenia group. The scientific basis for this threshold primarily comes from several large-scale retrospective analyses in China focusing on the Chinese population (20–22). Specifically, analysis of surgical specimens from Chinese gastric cancer patients found that the SMI cutoff value applicable to the Chinese population is 40.80 cm²/m² for males and 34.90 cm²/m² for females. When SMI falls below this threshold, the incidence of postoperative complications in patients significantly increases, effectively screening for high-risk patient groups with poorer prognoses.

**Figure 1:**
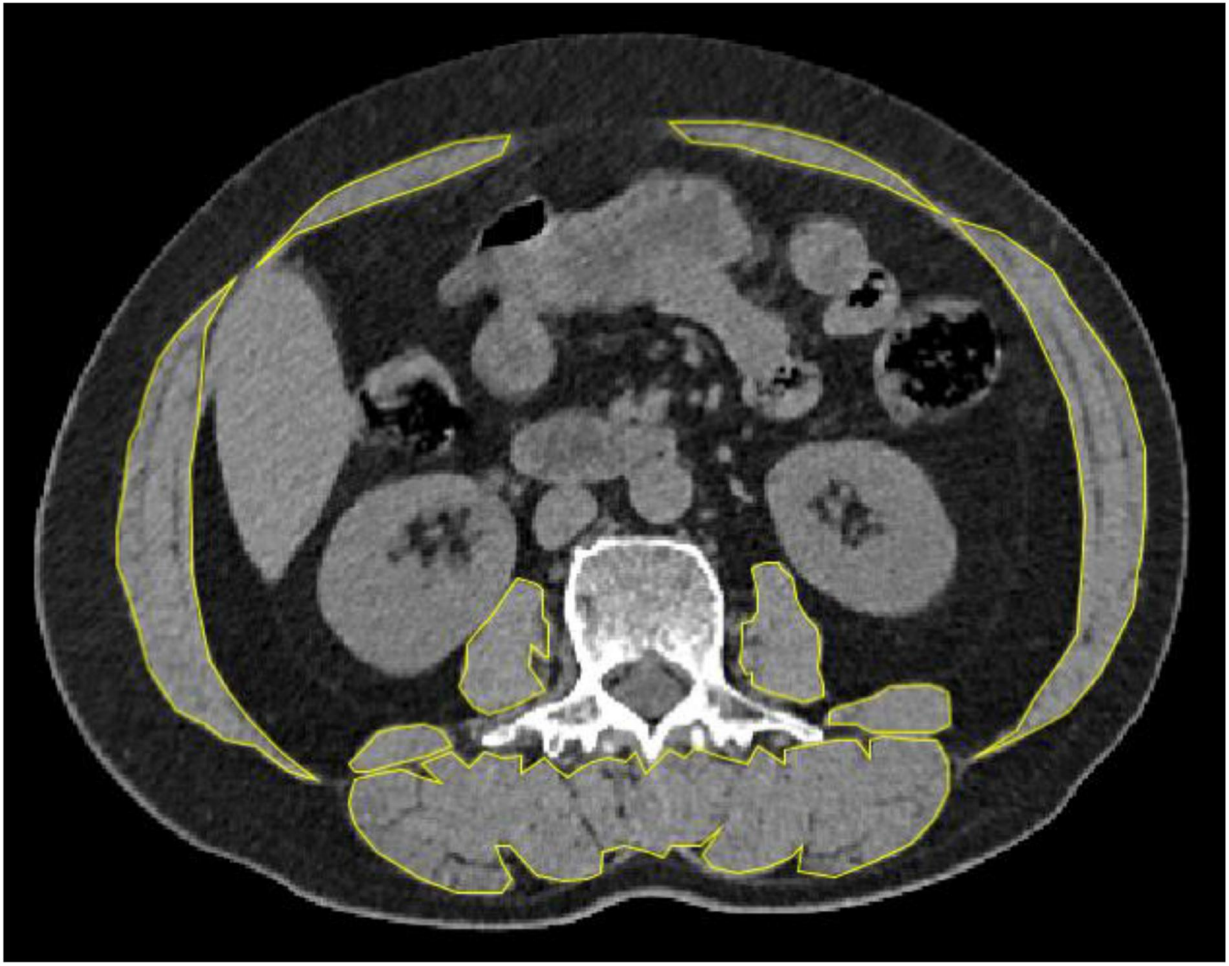
Schematic diagram of skeletal muscle contours at the L3 vertebral level.

All eligible patients underwent abdominal non-contrast CT scans after admission. The obtained CT image data in DICOM format were transferred and analyzed independently by two radiologists with over 5 years of experience in abdominal imaging diagnosis, who were blinded to the patients’ clinical information. The average of their measurements was taken. First, a clear transverse image at the midpoint level of the L3 vertebral body was selected. This level typically provides a good view of the major abdominal muscle groups. Subsequently, using ImageJ software, the outlines of all skeletal muscles at this level were manually traced, including the rectus abdominis, external and internal obliques, transversus abdominis, psoas major, quadratus lumborum, and erector spinae muscles (as shown in Figure 1). Finally, the software automatically calculated the total SMA in cm². To eliminate the influence of height on total muscle mass, the SMI was calculated using the formula: SMI (cm²/m²) = Total skeletal muscle area at L3 level (cm²)/height² (m²).

### 2.3 Data collection

General Clinical Data: Age, menopausal status, height, weight, BMI, smoking history, alcohol history, history of hypertension, history of diabetes, history of coronary heart disease.

Laboratory Test Indicators: Complete blood count, heart, liver, and kidney function tests, coagulation function, tumor markers. All blood samples were collected on the morning after admission under fasting conditions.

### 2.4 calculation of inflammatory markers

Based on complete blood count results, the following ratios were calculated:

PLR = Platelet count/Lymphocyte count.

NLR = Neutrophil count/Lymphocyte count.

MLR = Monocyte count/Lymphocyte count.

MHR = Monocyte count/High-density lipoprotein cholesterol.

PWR = Platelet count/White blood cell count.

HRR = Hemoglobin/Red blood cell distribution width.

Systemic Immune-Inflammation Index (SII) = Platelet count × Neutrophil count/Lymphocyte count.

Systemic Inflammation Response Index (SIRI) = Neutrophil count × Monocyte count/Lymphocyte count.

Aggregate Index of Systemic Inflammation (AISI) = Neutrophil count × Monocyte count × Platelet count/Lymphocyte count.

## 3 Statistical methods

Statistical analyses were performed using SPSS software (version 27.0). Normality of continuous variables was assessed using the Shapiro–Wilk test. Given the sample size of 263 cases, all continuous variables were initially tested; results indicated that all variables except body mass index (BMI) deviated from a normal distribution (*P* < 0.05). Consequently, data conforming to a normal distribution were expressed as mean ± standard deviation (x¯ ± SD) and compared using independent samples *t*-tests. Non-normally distributed data were presented as median (interquartile range) [M (P25, P75)] and analyzed using the Mann–Whitney *U*-test. Categorical variables were summarized as frequencies (percentages) and compared using the chi-square (*χ*²) test. Spearman correlation analysis was employed to evaluate the association between inflammatory markers and sarcopenia. Multivariate logistic regression analysis was conducted to identify independent risk factors for sarcopenia. A two-tailed *P*-value < 0.05 was considered statistically significant. Receiver operating characteristic (ROC) curves were constructed to evaluate the predictive efficacy of relevant indicators, with calculations for the area under the curve (AUC), optimal cut-off value, sensitivity, and specificity.

## 4 Results

### 4.1 Comparison of patient baseline characteristics

This study ultimately included 263 eligible middle-aged and elderly women with early-stage breast cancer. Based on the CT-measured L3-SMI cutoff value, there were 107 patients in the non-sarcopenia group and 156 patients in the sarcopenia group.

The comparison results of general clinical data and laboratory biochemical indicators between the two groups are detailed in Table 1. The results showed that regarding age, the mean age of patients in the sarcopenia group was significantly higher than that in the non-sarcopenia group (*P* < 0.05). Regarding menopausal status, the number of postmenopausal patients was higher in the sarcopenia group. In terms of anthropometric indicators, both body weight and BMI were significantly lower in the sarcopenia group than in the non-sarcopenia group (*P* < 0.05).

**Table 1:** Comparison of general data and biochemical indicators between the two groups .

Item	Non-sarcopenia group (*n* = 107)	Sarcopenia group (*n* = 156)	*t*-value	*P*-value
Age (years)	56.00 (47.75,62.00)	59.00 (52.00,67.00)	−2.615	0.009
Postmenopausal (n)	96	51	−1.970	0.049
Height (m)	1.58 (1.54,1.60)	1.60 (1.55,1.60)	−1.699	0.089
Weight (kg)	69.75 (64.00,75.00)	61.00 (55.00,69.00)	−5.535	<0.001
Hypertension (n)	55	51	−2.011	0.044
Diabetes (n)	18	16	−0.809	0.418
Coronary heart disease (n)	7	6	−0.411	0.681
BMI (kg/m^2^)	27.80 ± 3.38	24.83 ± 3.43	6.928	<0.001
Serum creatinine (*μ*mol/L)	57.60 (50.23,65.85)	55.70 (49.95,63.00)	−0.705	0.481
ALT (U/L)	19.00 (15.00,31.50)	17.00 (12.00,25.00)	−2.891	0.004
AST (U/L)	21.10 (18.00,29.00)	22.00 (18.50,26.50)	−0.086	0.932
Total protein (g/L)	75.60 (72.98,78.13)	74.90 (70.35,77.40)	−1.498	0.134
Albumin (g/L)	47.15 (44.73,49.10)	46.30 (44.20,48.25)	−1.645	0.100
Globulin (g/L)	28.65 (26.20,30.83)	27.90 (25.45,30.90)	−1.205	0.228
Prealbumin (mg/L)	269.25 (242.75,294.00)	254.00 (222.00,282.00)	−2.251	0.024
Cholinesterase (U/L)	9411.00 (8591.25,10327.25)	8396.00 (7196.50,9469.00)	−4.952	<0.001
White blood cell count (×10^9^/L)	6.02 (4.77,7.10)	5.60 (4.53,7.16)	−0.667	0.505
Neutrophil count (×10^9^/L)	3.95 (2.95,4.91)	4.79 (3.50,5.76)	−4.007	<0.001
Lymphocyte count (×10^9^/L)	1.58 (1.28,2.19)	1.29 (1.00,1.59)	−5.657	<0.001
Monocyte count (×10^9^/L)	0.30 (0.23,0.38)	0.28 (0.22,0.39)	−0.132	0.895
Eosinophil count (×10^9^/L)	0.08 (0.03,0.13)	0.07 (0.04,0.12)	−0.349	0.727
Hemoglobin (g/L)	133.00 (122.75,140.00)	128.00 (118.00,136.00)	−2.730	0.006
Red blood cell count (×10^12^/L)	4.40 (4.14,4.75)	4.29 (3.96,4.62)	−2.235	0.025
Platelet count (×10^9^/L)	229.50 (194.25,291.50)	248.00 (199.00,293.50)	−0.777	0.437
RDW-CV (%)	12.90 (12.50,13.50)	13.50(12.95,14.45)	−4.984	<0.001

Normality of continuous variables was assessed using the Shapiro–Wilk test. Most variables did not follow a normal distribution (*P* < 0.05) and are presented as median (interquartile range) [M (P25, P75)]. Body Mass Index (BMI) was normally distributed and is presented as mean ± standard deviation (x¯ ± SD). Comparisons between groups were performed using the independent samples *t*-test for BMI and the Mann–Whitney *U*-test for other variables.

Among the biochemical indicators reflecting liver function and nutritional status, alanine aminotransferase levels, cholinesterase levels, and prealbumin levels were lower in the sarcopenia group. The number of patients with comorbid hypertension was lower in the sarcopenia group, and the difference between the two groups was statistically significant. There was no statistically significant difference in the proportion of patients with comorbid diabetes or coronary heart disease between the two groups.

### 4.2 Comparison of blood inflammatory markers

We compared the complete blood count indicators and the calculated inflammatory markers between the two groups. The results are shown in Table 2. Compared with the non-sarcopenia group, neutrophil count was significantly higher in the sarcopenia group, while lymphocyte count was significantly lower. Concurrently, hemoglobin and red blood cell count were significantly lower in the sarcopenia group. RDW-CV, an indicator reflecting erythrocyte volume heterogeneity, was significantly higher in the sarcopenia group.

**Table 2:** Comparison of blood inflammatory markers between the two groups.

Item	Non-sarcopenia group (*n* = 107)	Sarcopenia group (*n* = 156)	*t* value	*P* value
PLR	156.62 (119.33,210.48)	181.91 (145.56,257.47)	−2.592	0.010
NLR	2.29 (1.82,2.95)	3.62 (2.66,5.28)	−7.484	<0.001
MLR	0.19 (0.15,0.27)	0.21 (0.17,0.30)	−2.328	0.020
MHR	0.18 (0.12,0.27)	0.17 (0.13,0.27)	−0.110	0.913
PWR	40.97 (30.53,50.76)	40.91 (32.60,54.67)	−0.767	0.443
HRR	1.02 (0.92,1.09)	0.95 (0.84,1.03)	−4.367	<0.001
SII	619.80 (406.69,913.27)	668.02 (454.42,1125.88)	−1.917	0.055
SIRI	0.74 (0.49,1.10)	0.83 (0.54,1.36)	−1.660	0.097
AISI	176.72 (111.03,274.41)	191.72 (113.19,378.45)	−1.438	0.150

Composite inflammatory markers calculated based on these basic indicators also showed significant differences. PLR, NLR, and MLR were all significantly higher in the sarcopenia group. In contrast, HRR, which combines anemia and inflammatory status, was significantly lower in the sarcopenia group.

### 4.3 Multivariate logistic regression analysis of breast cancer with sarcopenia

To further explore the independent risk factors for sarcopenia in breast cancer patients, variables with *P* < 0.05 in the univariate analysis were included in a multivariate binary logistic regression model. The results are shown in Table 3. Age, BMI, hemoglobin, and other variables were independent influencing factors for sarcopenia in middle-aged and elderly patients with early-stage breast cancer. Specifically, age, hemoglobin, and NLR were independent risk factors, with higher values associated with a greater risk of developing sarcopenia. BMI, red blood cell count, HRR, MLR, and RDW-CV were independent protective factors, with higher values associated with a lower risk of developing sarcopenia.

**Table 3:** Multivariate logistic regression analysis of factors associated with sarcopenia in the breast cancer cohort.

Item	*B*	*SE*	Wald *χ*^2^ value	*P* value	OR (95%*CI*)
Age	0.063	0.031	4.101	0.043	1.065 (1.002–1.131)
Postmenopausal	−0.243	0.576	0.178	0.673	0.784 (0.254–2.424)
Weight	0.075	0.042	3.300	0.069	1.078 (0.994–1.170)
BMI	−0.455	0.122	13.897	<0.001	0.634 (0.499–0.806)
ALT	0.006	0.008	0.541	0.462	1.006 (0.990–1.022)
Prealbumin	−0.001	0.002	0.712	0.399	0.999 (0.996–1.002)
Cholinesterase	0.000	0.000	1.332	0.248	1.000 (1.000–1.000)
Neutrophil	−0.186	0.176	1.108	0.293	0.831 (0.588–1.173)
Lymphocyte	0.055	0.129	0.183	0.699	1.057 (0.821–1.360)
Hemoglobin	0.145	0.072	4.068	0.044	1.156 (1.004–1.332)
Red Blood Cell	−0.063	0.021	9.049	0.003	0.939 (0.901–0.978)
RDW-CV	−1.256	0.596	4.448	0.035	0.285 (0.089–0.915)
PLR	−0.002	0.003	0.593	0.441	0.998 (0.992–1.003)
NLR	1.301	0.269	23.336	<0.001	3.671 (2.166–6.223)
MLR	−6.434	2.173	8.768	0.003	0.002 (0.000–0.114)
HRR	−25.032	10.020	6.242	0.012	0.000 (0.000–0.005)

### 4.4 Diagnostic value assessment of inflammatory markers for sarcopenia

**Figure 2:**
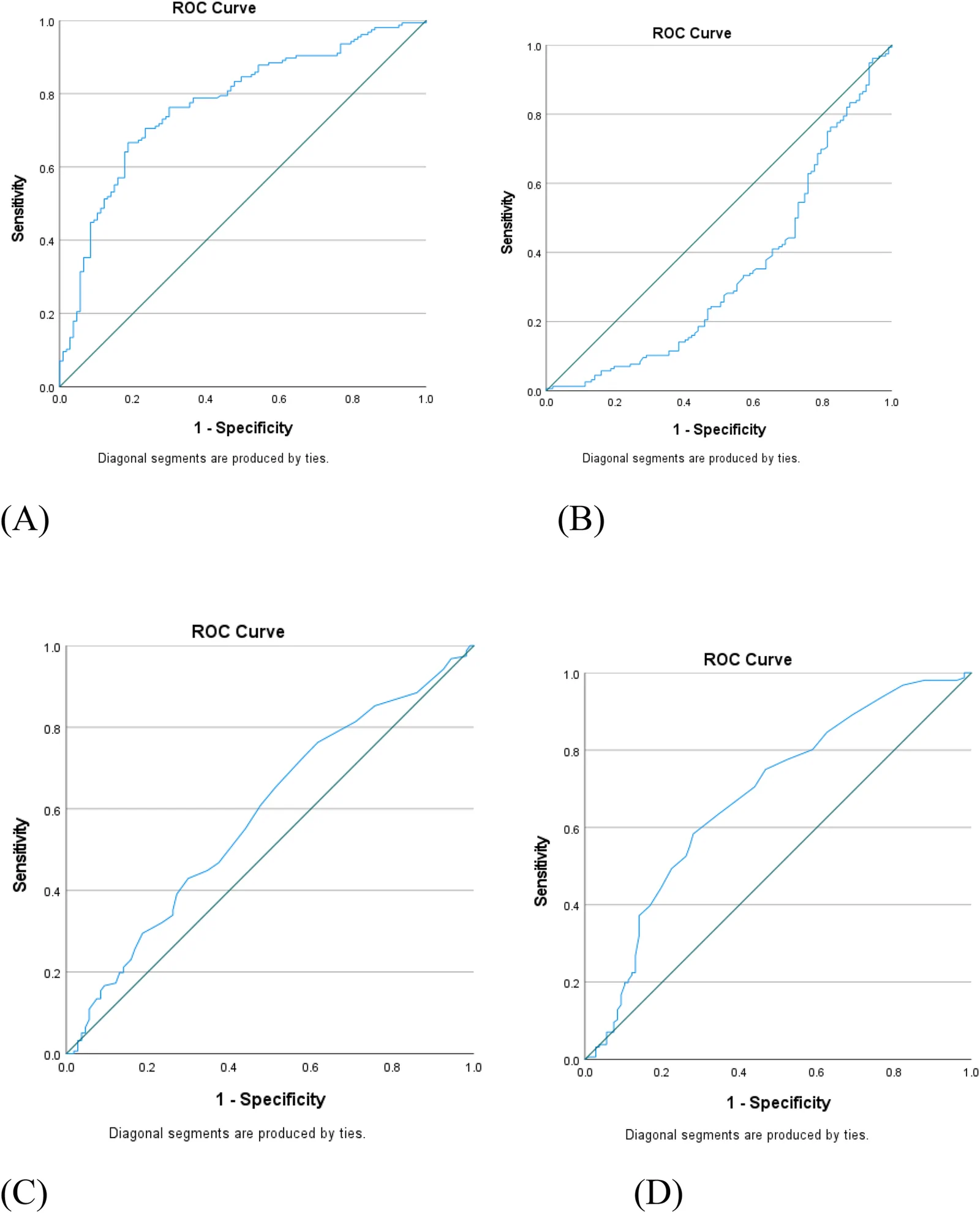
Roc curves of inflammatory markers for predicting sarcopenia. **(A)​** NLR (neutrophil-to-lymphocyte ratio); **(B)**​ MLR (monocyte-to-lymphocyte ratio); **(C)**​ RDW-CV (red blood cell distribution width); **(D)​** HRR (hemoglobin-to-red blood cell distribution width ratio).

To evaluate the potential clinical application of these easily accessible inflammatory markers in screening for sarcopenia, ROC curve analysis was performed on the independent influencing factors identified in the multivariate analysis: NLR, MLR, RDW-CV, and HRR. The results, as shown in Figure 2, indicate that only NLR demonstrated optimal diagnostic efficacy in predicting sarcopenia, with an AUC of 0.772 (95% CI: 0.714–0.829, *P* < 0.001), indicating moderate diagnostic value. Calculated based on the Youden index, the optimal cutoff value for NLR was 3.1427, with a sensitivity of 66.7% and a specificity of 81.3%. The results indicate that among the analyzed inflammatory markers, NLR is the most effective single indicator for predicting sarcopenia in middle-aged and elderly patients with early-stage breast cancer.

## 5 Discussion

This study is the first to systematically investigate the relationship between CT-imaging diagnosed sarcopenia and a series of peripheral blood inflammatory and nutrition-related markers in the specific population of middle-aged and elderly women with early-stage breast cancer. Our findings suggest that the occurrence of sarcopenia is closely associated with the body's systemic inflammatory state and nutritional imbalance, and multiple independent risk and protective factors were identified through multivariate analysis. Notably, we found that NLR, as a simple and economical biomarker, has good predictive value for sarcopenia, providing a potential effective tool for the early identification of high-risk patients in clinical practice.

Our study found that breast cancer patients with concurrent sarcopenia exhibited significant inflammatory characteristics, specifically manifested as elevated NLR levels. The increase in NLR, stemming from increased neutrophils and decreased lymphocytes, precisely depicts the imbalance in the immune microenvironment within cancer patients. Neutrophils, as the primary effector cells of innate immunity, can be activated in the tumor microenvironment and release large amounts of reactive oxygen species, proteolytic enzymes, and pro-inflammatory cytokines. These substances not only promote tumor progression but can also directly or indirectly induce catabolism in distant tissues (23). Concurrently, lymphocytes, particularly CD8⁺ T cells, are central to the body's adaptive anti-tumor immunity. A decrease in lymphocyte count, i.e., lymphopenia, is a marker of immunosuppression and immune exhaustion. This impaired immune function not only weakens immune surveillance of tumors but may also affect the body's ability to maintain tissue repair and metabolic homeostasis (24).

Persistent systemic inflammation, by activating key signaling pathways such as NF-*κ*B, can upregulate the activity of the muscle protein ubiquitin-proteasome system, a primary pathway leading to muscle atrophy (25). An elevated MLR reflects the activation of the monocyte-macrophage system. These cells are a major source of potent catabolic cytokines such as TNF-*α* and IL-6. These cytokines can directly act on muscle cells, induce insulin resistance, inhibit muscle protein synthesis, and promote its degradation, thereby exacerbating muscle wasting (26). The results of the multivariate analysis in this study also confirmed that lymphocyte count is an independent protective factor against sarcopenia, while MLR is an independent risk factor.

Another important finding of this study is that RDW-CV was significantly elevated and HRR was significantly lower in sarcopenia patients, and both were independent influencing factors for sarcopenia. RDW-CV is traditionally used for the differential diagnosis of anemia, but increasing evidence indicates that elevated RDW-CV is a sensitive marker for systemic inflammation and oxidative stress (27, 28). Under chronic inflammatory conditions, pro-inflammatory cytokines can interfere with iron metabolism and the responsiveness to erythropoietin, leading to impaired erythropoiesis, abnormal maturation, and shortened lifespan of red blood cells, thereby causing anisocytosis, i.e., elevated RDW-CV. Therefore, elevated RDW-CV not only reflects underlying nutritional deficiencies but, more importantly, reveals the inhibitory effect of inflammation on the hematopoietic system (29).

The observed independent association between decreased HRR and an increased risk of sarcopenia in this study suggests the potential protective role of this composite indicator in muscle metabolism regulation. HRR, as a novel biomarker, its core value lies in simultaneously reflecting the body's oxygen delivery capacity and the inflammatory status of the blood system. When HRR decreases, it often indicates relative hemoglobin insufficiency or a relative increase in red blood cell volume distribution width. Hemoglobin deficiency directly leads to insufficient oxygenation of muscle tissue, weakening muscle synthetic function; while increased RDW is often considered a reflection of the body's inflammatory response or malnutrition, factors that can accelerate muscle protein breakdown, leading to decreased muscle mass. Therefore, a lower HRR independently increases the probability of sarcopenia occurrence due to the higher risk of the body being in unfavorable conditions such as hypoxia, inflammation, and oxidative stress. This mechanism aligns with previous research conclusions on the negative correlation between HRR and geriatric frailty syndrome as well as osteoporosis risk, further supporting HRR as a valuable indicator for assessing the overall health status of the elderly (30). Currently, the gold standard for diagnosing sarcopenia relies on imaging assessment of muscle mass (e.g., CT, MRI, or DXA) combined with functional tests (e.g., grip strength, gait speed). However, these methods pose issues like radiation exposure or operational complexity in routine clinical practice. Therefore, developing simple and economical screening tools to identify high-risk populations for sarcopenia is particularly urgent. Our ROC curve analysis showed that NLR exhibits moderate and optimal diagnostic efficacy in predicting sarcopenia, with an optimal cutoff value of 3.1427, sensitivity of 66.7%, and specificity of 81.3%. Considering that complete blood count is a routine test for all inpatients, calculating NLR incurs no additional cost. These findings suggest a potential clinical application: For all newly diagnosed middle-aged and elderly early-stage breast cancer patients, NLR could be routinely calculated during the initial assessment. For patients with NLR above 3.1427, they could be considered at high risk for sarcopenia, and further precise evaluation is recommended, such as arranging body composition analysis or conducting simple functional tests. Once sarcopenia is diagnosed or highly suspected, multimodal interventions can be initiated before or early in anti-tumor treatment, including: (1) Nutritional support: Ensuring adequate protein and energy intake. (2) Exercise intervention: Focusing on resistance training combined with aerobic exercise to promote muscle protein synthesis and improve muscle function. (3) Potential anti-inflammatory strategies remain exploratory and should not currently be considered NLR-guided treatments. Whether interventions targeting inflammatory status according to NLR levels can improve muscle-related outcomes requires further prospective investigation.

This study has several notable strengths. First, it is one of the few studies focusing on the specific, relatively homogeneous population of middle-aged and elderly women with early-stage breast cancer, excluding the confounding effects of advanced disease cachexia and various treatment modalities. Second, we used CT-measured L3-SMI as the “gold standard” method for assessing muscle mass, ensuring the objectivity and accuracy of sarcopenia diagnosis. Finally, this study systematically evaluated a series of novel, easily accessible inflammatory markers and conducted comprehensive multivariate analysis and ROC curve evaluation, providing relatively reliable conclusions.

This study also has some inherent limitations. First, because of the retrospective cross-sectional design, this study can only demonstrate an association between inflammatory markers and sarcopenia rather than establish a causal relationship. Reverse causality and residual confounding cannot be excluded. In addition, as this was a single-center study conducted in a Chinese tertiary hospital, caution should be exercised when extrapolating these findings to other ethnic populations or healthcare settings. Therefore, prospective multicenter cohort studies are required to externally validate these findings before clinical implementation. Second, our sample size is relatively limited (*n* = 263), which may affect statistical power, especially in multivariate analysis where confidence intervals for some variables are wide. Third, the diagnosis of sarcopenia in this study was based solely on low muscle mass and did not incorporate assessments of muscle strength and physical function recommended by the European Working Group on Sarcopenia in Older People, which may result in an incomplete diagnosis of sarcopenia. Fourth, we were unable to perform subgroup analyses according to the molecular subtypes of breast cancer, as the biological behavior of different subtypes and host inflammatory responses may differ. Another important limitation is the absence of longitudinal follow-up. Consequently, we were unable to determine whether elevated NLR predicts clinically meaningful outcomes such as chemotherapy completion, treatment-related toxicity, postoperative complications, recurrence, or survival. Future studies with larger sample sizes should apply penalized regression techniques (e.g., LASSO or ridge regression), evaluate multicollinearity using variance inflation factors, and perform internal and external validation to improve model stability.

In summary, this study confirms that in middle-aged and elderly female patients with early-stage breast cancer, the occurrence of sarcopenia is significantly associated with a state of systemic inflammation and nutritional disorder characterized by elevated NLR, MLR, RDW-CV and decreased HRR. After multivariate adjustment, age, BMI, hemoglobin, red blood cell count, RDW-CV, NLR, MLR, and HRR were identified as independent influencing factors for sarcopenia. Among them, NLR, as a simple, non-invasive and economical blood marker, shows good predictive value for sarcopenia (AUC = 0.772) and may serve as an inexpensive preliminary screening indicator requiring further prospective validation. Although NLR demonstrated promising discriminatory ability, its clinical utility warrants confirmation in prospective, multicenter validation studies before routine clinical adoption. Regular monitoring of these inflammatory markers in clinical practice can help early identification and management of sarcopenia in breast cancer patients, thereby preventing the occurrence and progression of adverse events such as falls, fractures, prolonged hospital stay, increased risk of readmission, decline in physical function and disability, and increased mortality. This provides an important basis for implementing precise individualized supportive therapy and improving patients’ treatment tolerance and long-term prognosis.

## Data Availability

The original contributions presented in the study are included in the article/Supplementary Material, further inquiries can be directed to the corresponding authors.
